# Metamizole limits proliferation in chronic myeloid leukemia cells and triggers apoptosis via the bax/bcl-2/caspase-3 cascade

**DOI:** 10.1007/s12032-025-02842-x

**Published:** 2025-06-27

**Authors:** Erkan Maytalman, Dilara Nemutlu Samur

**Affiliations:** Department of Pharmacology, Faculty of Medicine, Alanya Alaaddin Keykubat University, Antalya, Türkiye

**Keywords:** Metamizole, K562, Chronic myeloid leukemia, Apoptosis, NSAID

## Abstract

Metamizole is a controversial non-steroidal anti-inflammatory drug because it may cause agranulocytosis usually in long-term use. It may reduce proliferation while increasing apoptosis in some cancer cells. In our study, the effects of increasing concentrations of metamizole on chronic myeloid leukemia (CML) cell line K562 were evaluated in terms of proliferation and apoptosis. K562 cells were cultured with 1,10,50,100 µM concentrations of metamizole in addition to the control group. The effect on cell proliferation was determined by MTT and analysis of mitotic cell counts. The apoptotic effects were analyzed by flow cytometry using Annexin V/Propidium iodide, ELISA for caspase-3 concentrations, and RT-qPCR for Bax–Bcl-2 mRNA expression levels. Evaluations were performed for 24 and 48 h of exposure. MTT assay revealed that metamizole limited the proliferation of cells at 10 µM concentration. Caspase-3 concentrations increased in cells exposed to concentrations of 50 µM and above. Flow cytometry results obtained using Annexin V/PI showed that especially 50 and 100 µM concentrations promoted apoptosis compared to the control. Bcl-2 mRNA expression was also significantly decreased at concentrations of 50 and 100 µM, while Bax mRNA expression was significantly increased only for 100 µM. Mitotic cell numbers also decreased with increasing concentrations. The known adverse effect of metamizole, agranulocytosis, suggests it may negatively affect cell proliferation. In this study, metamizole had both antiproliferative and pro-apoptotic effects on K562. The results of our study indicate that the synergistic effects of metamizole in the treatment of CML, especially in cases resistant to tyrosine kinase inhibitors, should be evaluated with further studies under in vitro and in vivo conditions.

**Supplementary Information:**

The online version contains supplementary material available at 10.1007/s12032-025-02842-x.

## Introduction

The production of prostanoids, which are the products of arachidonic acid metabolism, depends on cyclooxygenase (COX) activity. NSAIDs inhibit the biosynthesis of prostanoid subclasses such as prostaglandins, thromboxanes, and prostacyclins, by inhibiting COX. They are used in the treatment of various types of moderate pains, especially arthritis. Three isoforms of COX enzymes have been identified [1]. Metamizole is an analgesic and antipyretic drug that decreases prostaglandin synthesis by inhibiting COX-1 and COX-2 enzymes. However, its use remains highly controversial due to its adverse effect of causing agranulocytosis. Although it has been prohibited in many countries because of this side effect, it remains widely used in certain places and is available only by prescription in others [2]. Systematic reviews of the risks of NSAID-induced agranulocytosis and aplastic anemia first began in 1986 [3]. The incidence of metamizole-associated with agranulocytosis varies according to the studies. For example, in a study conducted in Germany between 2010 and 2013, the incidence of agranulocytosis and neutropenia associated with metamizole use was calculated as 1:1602. Similarly, in another study conducted in Europe, the incidence of agranulocytosis associated with metamizole use was reported as 0.96 cases/million people per year [2]. Although reviews based on case reports and case series suggest that metamizole poses a higher risk of causing agranulocytosis than other similar analgesics, the mechanisms involved have been little studied experimentally. Limited studies using cell cultures indicate that metamizole negatively impacts the proliferation and differentiation of myeloid cells. In this context, apoptotic effects were observed in undifferentiated granulocytic cells within the promyelocytic leukemia cell line (HL60) [4]. Using an in vitro colony-forming unit assay (CFU-MIX), it was found that metamizole suppressed the proliferation and differentiation of haematopoietic progenitor cells from healthy donors [5]. Moreover, it has been noted that metamizole increases the effectiveness of treatments for chronic myeloid leukemia [6] and P388 murine lymphocytic leukemia [7]. Furthermore, a limited number of studies have demonstrated the apoptotic and antiproliferative effects of metamizole in various other cancer cell lines [8–11].

Although metamizole has been shown to induce apoptosis in cells, the mechanisms are not clear. However, studies with other NSAIDs provide some insight. Nuclear factor-kappa B (NF-κB) is a key transcription factor that regulates the expression of numerous proinflammatory cytokines and anti-apoptotic proteins. Evidence has shown that increased COX-2 expression stimulates proliferation and invasiveness of OVCAR3 and SKOV3 (ovarian cancer cell lines) via the prostaglandin E2/NF-κB signaling pathway. The effects mediated by this signaling pathway were abolished in cells exposed to the selective COX-2 inhibitor celecoxib [12]. It has also been found that NSAID administration may reduce the NF-κB-mediated expression of inflammatory molecules in the early stages of experimental colorectal cancer [13]. In addition to NF-κB inhibition, the antiproliferative and apoptotic effects of NSAIDs include peroxisome proliferator-activated receptor gamma (PPARγ) activation and modulation of unfolded protein response (UPR) pathway components [14]. There is insufficient evidence about the contribution of COX-1 to these effects, but one study reported that sulindac, which can inhibit both COX-1 and COX-2, may cause NF-κB inhibition [13]. Metamizole and its metabolites are also nonselective COX inhibitors that can inhibit both COX-1 and COX-2 [15].

Programmed cell death, or apoptosis, is a strictly controlled biological process that is essential for preserving tissue homeostasis and eliminating damaged or aberrant cells. Cell shrinkage, chromatin condensation, DNA fragmentation, and caspase activation are among the defining morphological and biochemical features of apoptosis. Apoptosis evasion is a hallmark of tumor progression and treatment resistance in cancer biology. Consequently, inducing apoptosis in cancer cells is a key strategy in anticancer therapy development [16–18].

The results of studies with HL60 (as a myeloid-derived cell line), non-hematopoietic cell lines, hematopoietic cells in particular, and the side effect of the drug causing agranulocytosis prompted this study to investigate the effect of metamizole on chronic myeloid leukemia cells. This study aimed to evaluate the effects of metamizole on K562 cells at increasing concentrations by assessing proliferation and apoptosis markers.

## Methods

### Cell culture

The K562 cells (German Collection of Microorganisms and Cell Cultures, Braunschweig, Germany) available in our laboratory were retrieved from cryopreservation and initially expanded to achieve a sufficient quantity for the experiments. The cells were then divided into groups and cultured. Cell culture procedures were carried out using RPMI-1640 medium (Thermofisher, Gibco, UK) supplemented with 10% FBS (Thermofisher, Gibco, UK), 100 U/ml penicillin, and 100 µg/mL streptomycin (Thermofisher, Gibco, UK) under the standard conditions of 37 °C, 5% CO₂, and a humidified atmosphere (Thermo Scientific, İ160, Germany).

### Proliferation/cytotoxicity analyses

To determine the IC₅₀ value and assess the effects of increasing concentrations of metamizole on cell proliferation, an MTT assay was performed. The cells were cultured in 8 wells for each group using a 96-well plate for the experiment and this process was repeated three times. Experimental groups were treated with 1, 10, 50, 100, and 200 µM of metamizole (Sigma-Aldrich, USA), and their effects were evaluated at 24 and 48 h in comparison with the control group. For each group, cells were seeded into 96-well plates at a density of 1 × 10^4^ cells per 100 µL per well. At the end of the incubation period, 10 µL of MTT (3-(4,5-dimethylthiazol-2-yl)-2,5-diphenyltetrazolium bromide) (Sigma-Aldrich, UK) solution prepared at a concentration of 5 mg/mL was added to each well. The plate was then incubated for an additional 4 h to allow for formazan crystal formation. The resulting formazan crystals were dissolved using dimethyl sulfoxide (Carlo Erba, France), and absorbance values were measured at 570 nm using a multimode plate reader (BioTek Synergy H1, USA).

### The analyses of viability and apoptosis

These experiments were conducted on control and treatment groups exposed to 1, 10, 50, and 100 µM concentrations of metamizole.

### Annexin V/PI flow cytometry analyses

The proportions of apoptotic cells were determined using FITC-annexin V and propidium iodide (PI) staining (BD Pharmingen, FITC Annexin V Apoptosis Detection Kit, USA). This assay allowed for the identification of early apoptotic, late apoptotic, necrotic, and viable cell populations. For this experiment, the cells were washed with PBS and then resuspended in 1 mL 1X binding buffer. Then, 100 μL of the cell suspension was transferred to each tube. 5 μL FITC-Annexin V and 5 μL propidium iodide (PI) were added to the cell solutions. These samples were gently vortexed and then incubated for 15 min at room temperature in the dark. Afterward, 400 μL of 1X binding buffer was added to each tube. Flow cytometry (BD FACSCanto II, USA) analyses were performed using 1 × 10⁶ cells collected from each group. Each group was cultured in four biological replicates, with two technical replicates for flow cytometric analysis. Data acquisition and analyses were carried out using FACS DIVA software (BD Biosciences, USA).

### Assessment of Bax and Bcl-2 mRNA Expression via RT-qPCR

For this purpose, three replicate cell cultures were performed for each group. Following cell counting using the automated cell counter (BioRad TC20, Singapore), 2 × 10⁶ cells from each group were used for total RNA isolation. RNA was extracted using a commercial isolation kit (Norgen Biotek, Canada). RNA concentrations were determined with a multimode reader (BioTek Synergy 2, USA). Complementary DNA (cDNA) synthesis was performed using a ready-to-use cDNA synthesis kit (Thermo Scientific, Germany) in a thermal cycler (Applied Biosystems, SimpliAmp, Thermo Fisher Scientific, USA). To evaluate mRNA expression changes among groups, RT-qPCR analyses were conducted using the LightCycler 96 System (Roche Diagnostics, Germany). Analyses were performed in triplicate for each sample and primer. Each primer was tested in three technical replicates within the same experiment. Fluorescence emitted by SYBR Green (LightCycler 480 SYBR Green I Master Roche Diagnostics, Germany), which binds to double-stranded DNA and increases with each amplification cycle, was monitored in real time. The threshold cycle (Ct) values were used for quantitative analyses of gene expression. The primer sequences used for Bax and Bcl-2 gene expression analyses were as follows (Microsynth AG, Switzerland):

Bax:

- F: 5′-GGGTGGTTGGGTGAGACTC-3′
- R: 5′-AGACACGTA AGG AAA ACGCAT TA-3′

Bcl-2:

- F: 5′-TCCGCATCAGGA AGGCTA GA-3′
- R: 5′-AGGACCAGGCCTCCAAGC T-3′

β-Actin (Housekeeping):

- F:5′-CACCATTGGCAATGAGCGGTTC-3′
- R: 5′-AGGTCTTTGCGGATGTCCACGT-3′.

### Measurement of caspase-3 activity via enzyme-linked immunosorbent assay (ELISA)

Caspase-3 concentrations were measured using a human caspase-3 ELISA kit (Sunred, Shanghai, China). 1 × 10⁶ cells/mL were used for each group. After subjecting the cells to freeze–thaw cycles, the resulting supernatants were used for analyses. All procedures were carried out in accordance with the manufacturer’s instructions. Following the completion of the assay, absorbance was measured at 450 nm using a multimode plate reader (BioTek Synergy H1, USA). Caspase-3 concentrations were then calculated based on the standard curve. ELISA analyses were performed in triplicate for each of three independent cultures.

### Assessment of cells in the mitotic phase

Cells were cytocentrifuged onto glass slides and stained with Giemsa solution, which is suitable for haematologic cell types. The cell slides were fixed with methanol for 3 min after drying. After washing with water, a 1:9 dilution of giemsa stain was added on the slides and stained for 20 min. After the staining period, the slides were washed again and dried. The stained slides were examined under a light microscope (Carl Zeiss, Axio Lab.A1, Germany), and the proportion of cells undergoing mitosis was determined. Cells that were microscopically distinguishable in the prophase, metaphase, anaphase, or telophase stages of mitosis were classified as mitotic. For each group, a total of 500 cells were counted per slide to evaluate the percentage of cells in the mitotic phase.

### Statistical analyses

Absorbance values obtained from all samples in the MTT assay were converted into percentages, with the control group set at 100%. Data were analyzed using the Shapiro–Wilk test, which revealed that the distributions were not normal across groups. For gene expression analyses, threshold cycle (Ct) values were normalized to the reference gene β-Actin, and fold changes were calculated using the 2^(−∆∆Ct)^ method. Comparisons between groups were evaluated using the Kruskal–Wallis test, followed by Dunn’s post hoc correction. All statistical analyses were performed using GraphPad Prism version 9.0.0 (Dotmatics, Insight Partners, NY, U.S.A.). Additionally, for the evaluation of Bax and Bcl-2 gene expression, fold changes greater than two compared to the control group were considered physiologically meaningful.

## Results

### Proliferation/cytotoxicity analyses

**Fig. 1:**
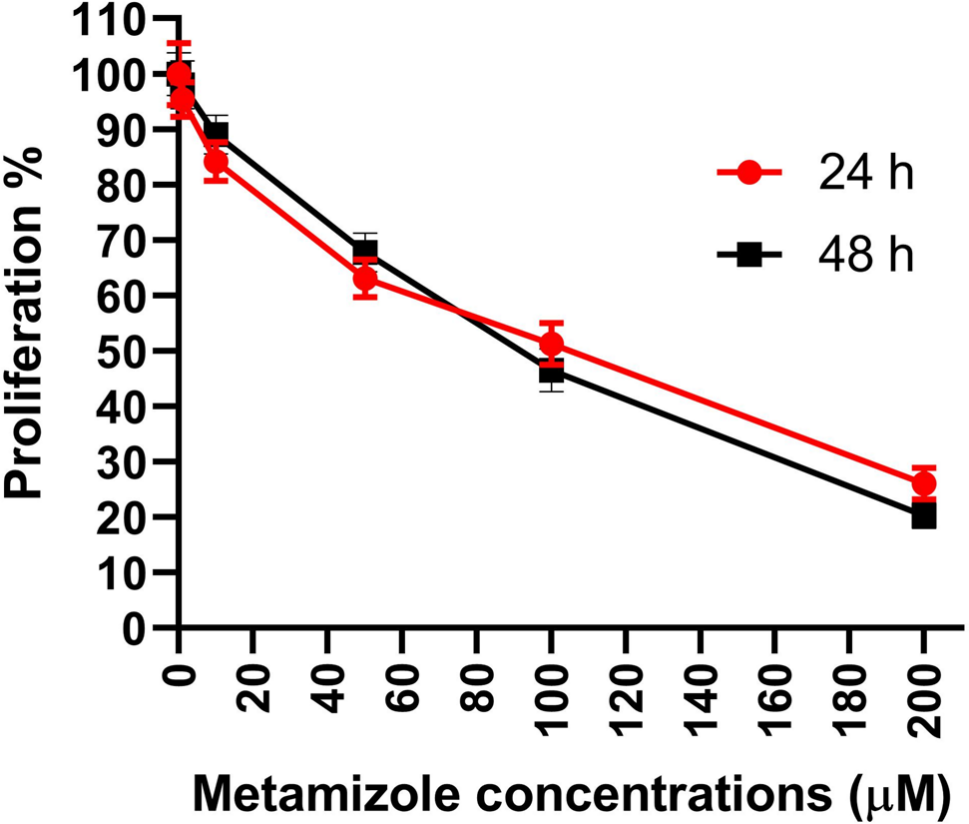
Concentration–response curves after 24 and 48 h of metamizole exposure

According to MTT assay results, significant reductions in cell proliferation were observed at 10, 50, 100, and 200 µM concentrations following both 24- and 48-h exposures. Additionally, time-dependent differences were detected between 24- and 48-h treatments within each concentration group. An increase in proliferation was noted at 1, 10, and 50 µM at 48-h, whereas 100 and 200 µM concentrations led to a reduction compared to the 24-h exposure (Table 1). The IC₅₀ values of metamizole were calculated as 117.5 ± 1.13 µM for 24 h and 111.2 ± 2.26 µM for 48 h. The change in effect is seen in Fig. 1, which shows the concentration–response curve.

**Table 1:** Changes in the proliferation capacity of K562 cells exposed to different concentrations of metamizole for 24 and 48 h

Concentrations (µM)	24 h		48 h		Significant differences (24 vs. 48)
	% Mean ± SD	Significant differences (vs. control)	% Mean ± SD	Significant differences (vs. control)	
Control	100.0 ± 5.633	–	100.0 ± 3.902	–	ns
1	95.37 ± 3.084	ns	98.08 ± 4.352	ns	*p = 0.0180
10	84.22 ± 3.547	**p = 0.0074	89.03 ± 3.528	*p = 0.0329	****p < 0.0001
50	63.10 ± 3.364	****p < 0.0001	67.75 ± 3.546	****p < 0.0001	***p = 0.0002
100	51.28 ± 3.766	****p < 0.0001	46.55 ± 3.881	****p < 0.0001	****p < 0.0001
200	26.10 ± 2.839	****p < 0.0001	20.28 ± 2.278	****p < 0.0001	****p < 0.0001

Statistical analyses was performed using the Kruskal–Wallis test followed by Dunn’s post hoc correction. Data are shown as mean + SD

### Annexin V/PI flow cytometry analyses

**Fig. 2:**
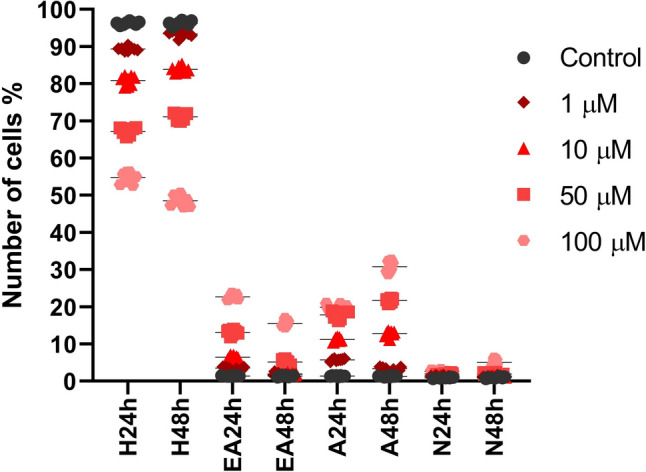
Dot plot distribution of healthy (H), early apoptotic (EA), apoptotic (A), and necrotic (N) cells after 24 and 48 h of metamizole exposure based on flow cytometry data

A significant decrease in the percentage of viable cells was observed at 50 and 100 µM concentrations compared to the control group following both 24- and 48-h exposures. In contrast, the same concentration groups showed a significant increase in early apoptotic, late apoptotic, and necrotic cell populations. In addition, certain high-concentration groups demonstrated significant differences not only compared to the control but also in relation to the 1 and 10 µM treatment groups. Notably, distinct differences were observed in the proportions of early and late apoptotic cells between the 24- and 48-h exposure periods. Specifically, early apoptotic cells were more prominent at 24 h, whereas their proportion decreased at 48 h, accompanied by a corresponding increase in late apoptotic cells (Table 2) (Suppl. Figure). Additionally, the dotplot distribution of healthy, early apoptotic, apoptotic, and necrotic cells according to flow cytometry results is shown in Fig. 2.

**Table 2:** Flow cytometric analyses of apoptotic cells using Annexin V/Propidium iodide staining

Groups		Cont	1 µM	10 µM	50 µM	100 µM
Healthy cells (%)	24 h	96.29 ± 0.44	89.35 ± 0.54	80.85 ± 1.25	67.13 ± 0.9 ***p	54.74 ± 1.46 ****p/^^^*p*
	48 h	96.28 ± 0.67	93.43 ± 0.89 °°°*p*	83.89 ± 0.83 °°°*p*	71.13 ± 0.85 ***p/°°°*p*	48.58 ± 1.45 ****p/^^^*p*/°°°*p*
Early apoptotic cells (%)	24 h	1.41 ± 0.11	3.54 ± 0.29	6.44 ± 0.44	13.13 ± 0.66 ***p	22.68 ± 0.66 ****p/^^^*p*
	48 h	1.4 ± 0.21	1.988 ± 0.39 °°°p	1.91 ± 0.27 °°°*p*	5.18 ± 0.56 ***p/°°°*p*	15.53 ± 0.77 ****p/^^*p*/^♦♦^*p*/°°°*p*
Apoptotic cells (%)	24 h	1.39 ± 0.11	5.76 ± 0.3	11.24 ± 0.47	17.81 ± 0.96 ***p	19.9 ± 0.77 ****p/^^^*p*
	48 h	1.34 ± 0.17	3.38 ± 0.41 °°°*p*	12.79 ± 0.77 °°*p*	21.74 ± 0.59 ***p/°°°*p*	30.83 ± 1.47 ****p/^^^*p*/°°°*p*
Necrotic cells (%)	24 h	0.91 ± 0.12	1.35 ± 0.21	1.48 ± 0.1	1.93 ± 0.21 ***p	2.688 ± 0.3834 ****p/^^*p*/^♦^*p*
	48 h	0.99 ± 0.2	1.21 ± 0.24	1.41 ± 0.16	1.96 ± 0.25 **p	5.075 ± 0.7686 ****p/^^*p*/^♦^*p*/°°°*p*

Data are shown as mean ± SD

Statistical analysis was performed using the Kruskal–Wallis test, followed by Dunn’s post hoc correction. Kruskal Wallis – Dunn’s correction

* vs. cont.; ^ vs. 1 µM; ^♦^ vs. 10 µM; ° 48 h vs. 24 h

*^/^^^/♦/^°*p* < 0.05; **^/^^^^/♦♦/^°°*p* < 0.01; ***^/^^^^^/♦♦♦/^°°°*p* < 0.001; ****^/^^^^^^/♦♦♦♦/^°°°°*p* < 0.0001

### Measurement of caspase-3 activity via enzyme-linked immunosorbent assay (ELISA)

**Fig. 3:**
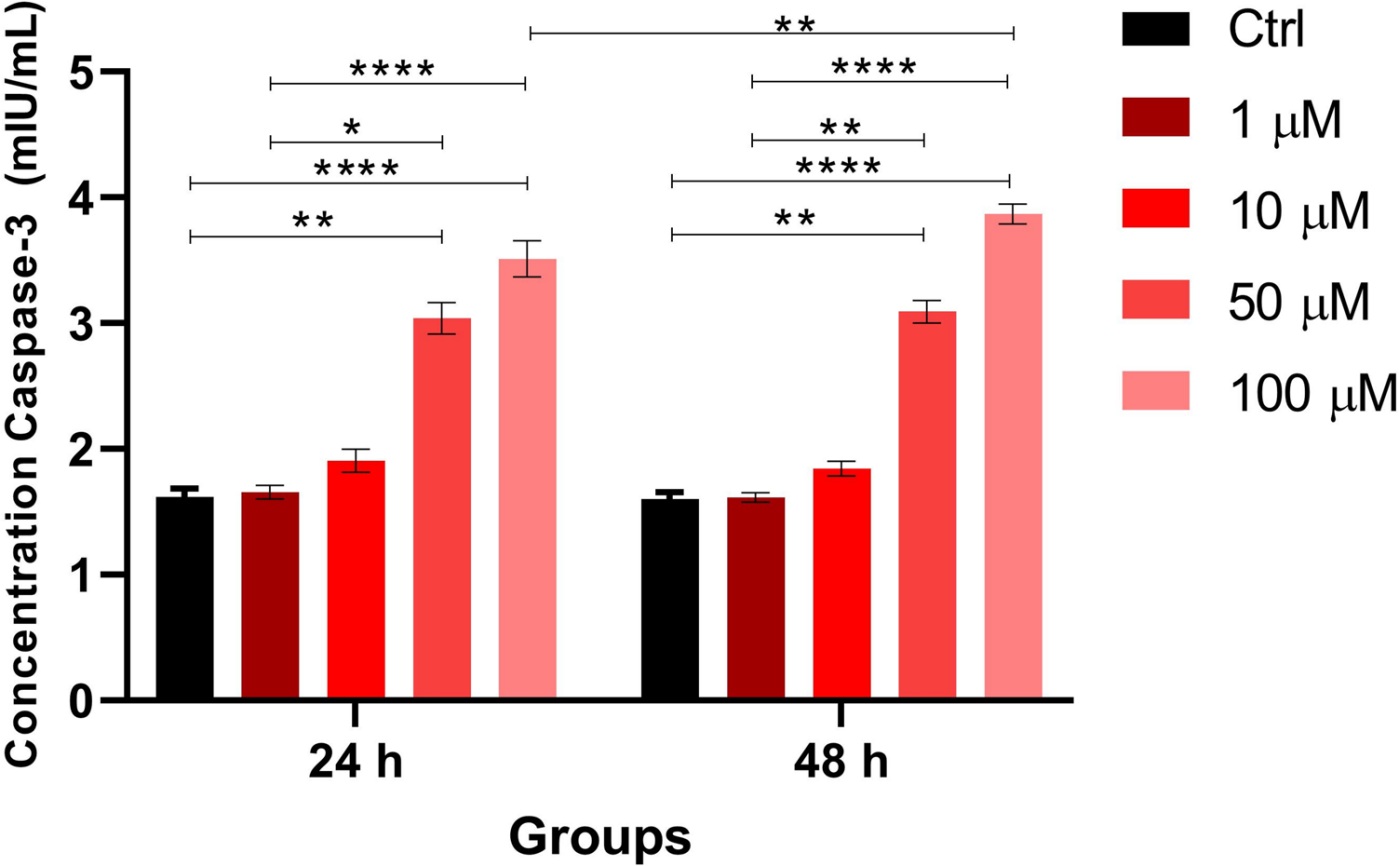
Caspase-3 concentrations at 24- and 48-h exposures as determined by ELISA. Three replicates from each of the three independent cultures were studied. Statistical analysis was performed using the Kruskal–Wallis test, followed by Dunn’s post hoc correction. **p < 0.01, ***p < 0.001, ****p < 0.0001. Data are shown as mean ± SD

Significant increases in caspase-3 concentrations were observed at both 24- and 48-h exposures in the 50 µM (24 h: 3.04 ± 0.12; 48 h: 3.09 ± 0.09 mIU/mL) and 100 µM (24 h: 3.51 ± 0.14; 48 h: 3.87 ± 0.08 mIU/mL) treatment groups compared to the control (24 h: 1.62 ± 0.07; 48 h: 1.60 ± 0.05 mIU/mL) and 1 µM (24 h: 1.66 ± 0.05; 48 h: 1.61 ± 0.04 mIU/mL) groups. A statistically significant difference between the 24- and 48-h exposures was observed only at the 100 µM concentration (Fig. 3).

### Assessment of Bax and Bcl-2 mRNA expression via RT-qPCR

**Fig. 4:**
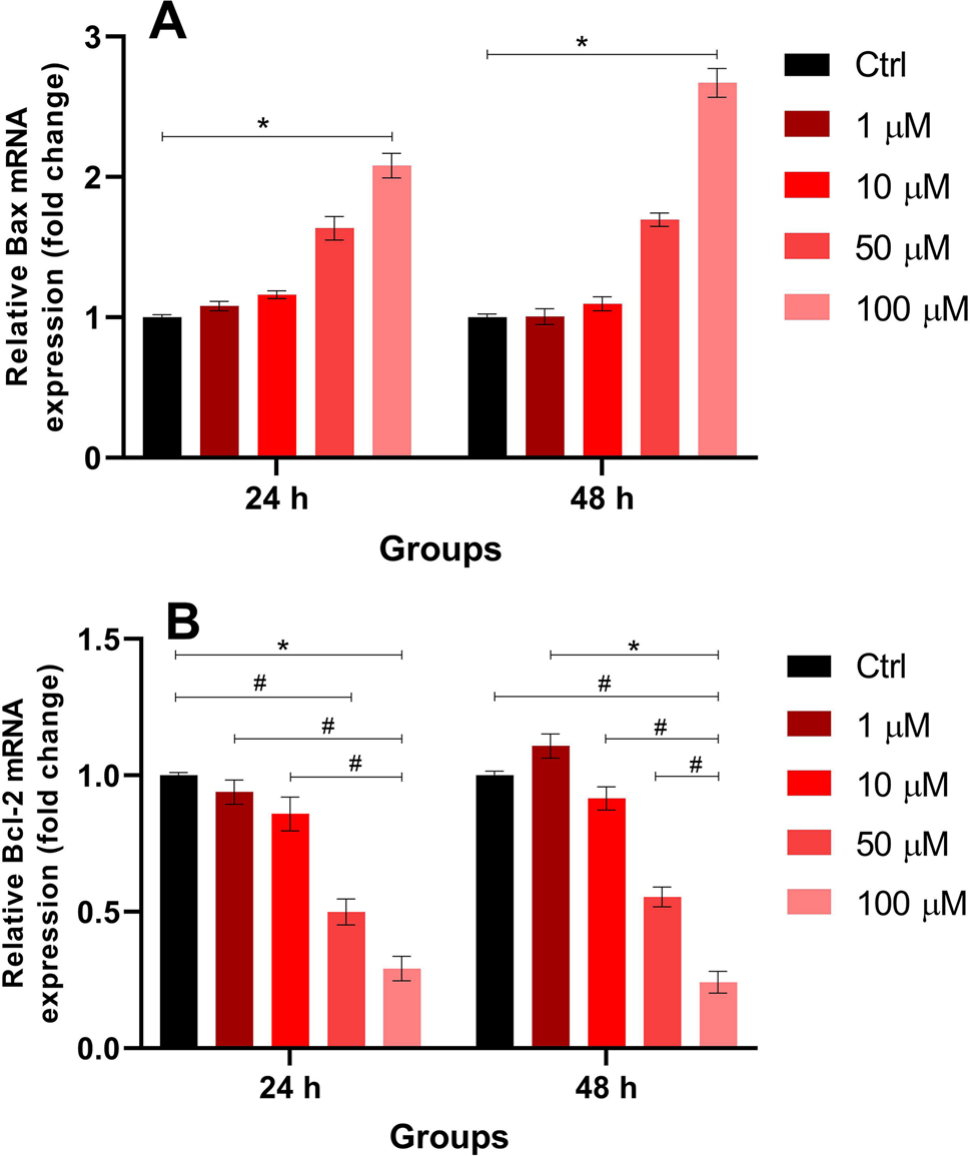
Relative mRNA expression levels of Bax (**A**) and Bcl-2 (**B**) at 24- and 48-h exposures as determined by RT-qPCR. Statistical analysis was performed using the Kruskal–Wallis test, followed by Dunn’s post hoc correction. *p < 0.05; #: fold change greater than 2, considered physiologically significant. Data are shown as mean ± SD

An increase in Bax mRNA expression was observed only at the 100 µM concentration (fold change 24 h: 2,08 ± 0.09; 48 h: 2,67 ± 0.1) compared to the control (fold change 24 h: 1 ± 0.03; 48 h: 1 ± 0.06). Bcl-2 mRNA expression showed a significant decrease at 100 µM following 24-h exposure (fold change: 0.29 ± 0.04) compared to the control (1 ± 0.01) and at 48 h (fold change: 0.24 ± 0.04) compared to the 1 µM group (1.11 ± 0.02). Additionally, more than twofold changes in Bcl-2 mRNA expression were detected between certain groups, which were considered physiologically significant (Fig. 4). Additionally, bax/bcl-2 ratios were calculated based on fold changes and are presented in Table 3.

**Table 3:** Ratio of fold changes for bax and bcl-2 after 24 and 48 h of exposure

Bax/Bcl-2 ratio					
	Control	1 µM	10 µM	50 µM	100 µM
24 h	1 ± 0.03	1.15 ± 0.06	1.36 ± 0.13	3.28 ± 0.15	7.21 ± 0.82*
48 h	1 ± 0.03	0.91 ± 0.07	1.2 ± 0.09	3.06 ± 0.14	11.24 ± 1.87^**#**^

Statistical analysis was performed using the Kruskal–Wallis test, followed by Dunn’s post hoc correction. *p < 0.05 versus control-24 h; ^**#**^*p* < 0.05 versus 1 µM concentration-48 h. Data are shown as mean ± SD

### Assessment of cells in the mitotic phase

**Fig. 5:**
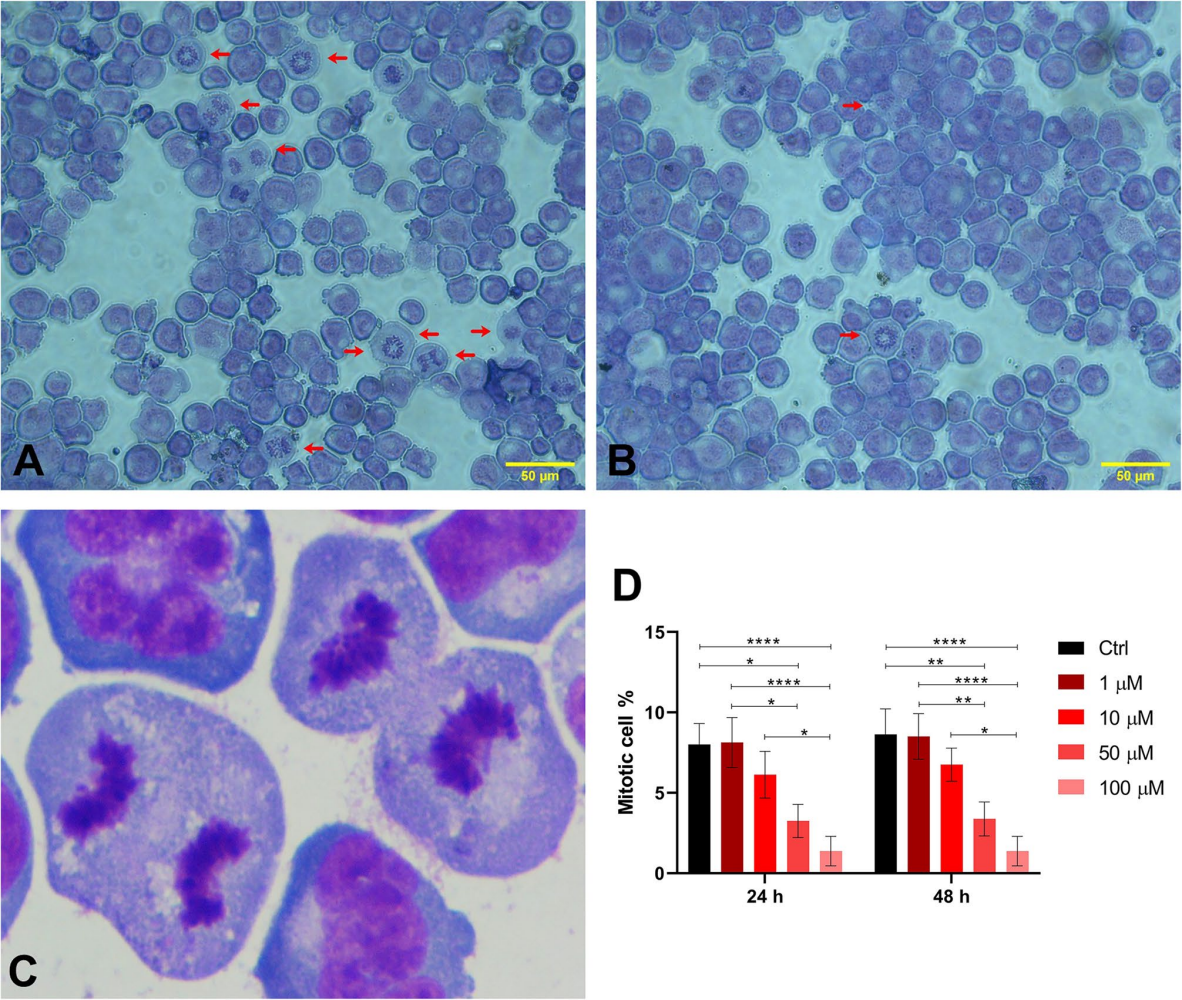
**A** Cells in mitotic phase at 1 µM concentration; **B** cells in mitotic phase at 100 µM concentration; **C** high-magnification view of mitotic cells (× 100) on giemsa stained slides. **D** Comparison of mitotic cell ratios across experimental groups. Statistical analyses were performed using the Kruskal–Wallis test followed by Dunn’s post hoc correction. *p < 0.05, **p < 0.01, ***p < 0.001, ****p < 0.0001. Data are shown as mean ± SD

Microscopic analyses of Giemsa-stained preparations revealed a decrease in the number of cells in mitotic phases (prophase, metaphase, anaphase, and telophase) with increasing concentrations of metamizole. In the control group, the percentage of cells in mitosis was 8 ± 1.3% at 24 h and 8.63 ± 1.6% at 48 h. Notably, a dramatic reduction in the proportion of mitotic cells was observed at both exposure times in the 50 µM (24 h: 3.25 ± 1.04%; 48 h: 3.38 ± 1.06%) and 100 µM (24 h and 48 h: 1.38 ± 0.92%) treatment groups (Fig. 5).

## Discussion

The mechanisms underlying metamizole-induced agranulocytosis and its effects on cancer cells are not yet fully understood. Evidence regarding the effect of metamizole on hematopoiesis is mainly limited to case reports. One of the reports identified that the side effect may be explained by a direct toxic effect on hematopoietic progenitor cells. The findings indicate that granulopoiesis is selectively suppressed, while the generation of other blood cell lineages remains unaffected [19]. Another report based on bone marrow biopsy examinations suggests that the drug may inhibit granulocyte maturation at the myelocyte stage, potentially leading to the development of agranulocytosis [20]. In an in vitro study, the combination of metamizole’s metabolite, *N*-methyl-4-aminoantipyrine (MAA) (100 µM), and hemin (12.5 μM) was found to decrease the colony-forming capacity of immature myeloid cells derived from umbilical cord blood. In the study, the toxicity of metamizole on the promyelocytic cell line HL60 was also shown to be hemin dependent [21]. Recent studies using hematopoietic stem cell-enriched product samples obtained from healthy allogeneic transplant donors showed that metamizole at 10 and 100 μM concentrations resulted in a decrease in the number of erythroid (CFU-E) and granulocyte–macrophage (CFU-GM) colonies. Additionally, immunofluorescence staining revealed a reduction in granulocyte differentiation at the 100 μM concentration [5]. In a study conducted on healthy volunteers treated with metamizole, a daily dose of four tablets over seven days did not significantly alter total leukocyte counts; however, it caused reductions in erythrocyte and platelet counts, as well as hemoglobin levels [22]. Some studies have suggested that immunological mechanisms [4] and genetic factors may contribute to these adverse effects. [4, 23]. Penicillin allergy was a postulated risk factor for leukopenia caused by metamizole in a case–control study among a European population in Switzerland [24]. A genetic study identified two loci on chromosome 9, rs55898176 and rs4427239, as potential candidates involved in metamizole-related adverse effects. The latter locus is located within the SVEP1 gene, which is known to play a role in hematopoiesis [25].

Reports that indicate metamizole may inhibit the development of myeloid-stage cells, along with limited evidence from cancer cell line studies [8–11], directed us to focus on the K562 cell line, particularly due to its myeloid origin. Building on a previous pilot study conducted with concentrations ranging from 1 to 20 μM, we redesigned the experimental protocol to cover a broader range (1–200 μM) and presented comprehensive apoptosis data in the current study.

The first studies investigating the effects of metamizole on myeloid-derived cancers date back to the early 1990s. In an in vitro cytotoxicity experiment using cells obtained from patients with chronic myeloid leukemia (CML), the results showed that metamizole alone caused significant inhibition, and its combination with adriamycin caused an irreversible cytotoxic effect [6]. In another study, in P388 lymphocytic leukemia murine models, the addition of metamizole to the treatment of both drug-sensitive and drug-resistant disease models enhanced the antitumor efficacy of mitoxantrone or doxorubicin [7, 26]. These early studies were limited in methodology due to the technological constraints of that time. In this study, we extensively investigated the effects of metamizole on K562 cells using flow cytometry, ELISA, and RT-qPCR.

Analysis of annexin V/PI-labeled cells by flow cytometry is a very convenient method for quantifying the proportions of healthy (Annexin V⁻/PI⁻), early apoptotic (Annexin V⁺/PI⁻), apoptotic (Annexin V⁺/PI⁺), and necrotic (Annexin V⁻/PI⁺) cells [27, 28]. Metamizole and its active metabolite MAA were found to significantly inhibit the proliferation of human pancreatic cancer cell lines PaTu 8988t and Panc-1, both alone and in combination with paracetamol, as assessed by BrdU-based proliferation and flow cytometry assays [8]. Shao et al. [9] demonstrated that metamizole negatively affects the proliferation of A549 lung cancer cells using the Sulforhodamine B assay. Additionally, flow cytometry analyses with PI staining revealed that 48-h exposure to metamizole induced G1 phase cell cycle arrest. Bundscherer et al. [10] reported that the proliferation of colorectal cancer cell lines HT-29 and SW 480 was not affected by metamizole or its metabolite MAA, even at high concentrations, according to BrdU analyses. However, Annexin V/PI flow cytometry and western blot results for caspase-3, -8, and -9 showed that the active substances induced apoptosis in HT-29 cells. In MG-63 osteoblast-like osteosarcoma cells, Hoechst/propidium iodide staining indicated that metamizole induced apoptosis, particularly under culture conditions designed to promote osteogenic differentiation. Furthermore, 24-h incubation with 1 and 10 μM concentrations of metamizole significantly reduced the expression of key osteoblast phenotype markers, including CD80, CD86, and HLA-DR. In addition, a decrease in both phagocytic activity and osteogenic differentiation capacity was observed. The study also reported comparable but variable effects with other NSAIDs such as dexketoprofen, ketorolac, and acetylsalicylic acid [11]. Contrary to these findings, some studies have reported that metamizole may exert a protective effect in certain osteosarcoma cell lines, such as canine D-17 and human U-2 OS, by reducing the antitumor activity of risedronate sodium [29]. A similar cytoprotective effect was observed in HL-60, Jurkat, and Raji cell lines exposed to UV irradiation, arachidonic acid, and cycloheximide treatments at metamizole concentrations below 300 μM. However, cytotoxic effects were observed at higher concentrations [30]. Our study presents a comprehensive analysis based on a broad range of techniques.

The findings of this study are consistent with previous reports of metamizole-induced apoptosis in other cell lines. However, it also reveals distinct findings not reported in previous studies. Interestingly, the early apoptotic cell population detected at 24 h was largely replaced by apoptotic cells at 48 h, suggesting progression through the apoptotic cascade over time. Additionally, at lower concentrations, the proliferative activity appeared to increase at 48 h compared to 24 h, possibly indicating a time-dependent reduction in drug efficacy following a single-dose exposure. The cells were also visualized in the mitotic stage. Significant decreases in the number of mitotic cells were detected at increasing concentrations. These results suggest that metamizole does not cumulatively inhibit proliferation but instead slows it in a concentration-dependent manner. It was observed that the proportion of early apoptotic cells decreased in 48 h and the number of apoptotic cells increased, especially starting from a concentration of 10 µM. These results may provide a clue to explain why agranulocytosis usually occurs with long-term use of the drug. However, the therapeutic effects of antineoplastics in cancer treatments depend on cell cycles. Previous studies have shown that the use of metamizole together with antineoplastics contributes to the therapeutic effect [6, 7, 26]. The stepwise apoptosis of a certain proportion of cells suggests that the effect of metamizole may be dependent on cell cycle and concentration. The mechanisms may also include different toxic effects of metamizole. Studies in both hematopoietic cells and the myeloid HL60 cell line have indicated that increased hemin levels may contribute to metamizole-induced cytotoxicity [21, 31]. Ferroptosis, a non-apoptotic cell death regulated by phospholipid hydroperoxide glutathione peroxidase 4 (GPX4), may explain the iron-related effects of metamizole. Inhibition of GPX4, which usually reduces levels of lipid reactive oxygen species (ROS), compromises the cell’s antioxidant defenses and ultimately leads to iron-dependent oxidative cell death [32]. The discovery of GPX4 inhibitors represents a promising therapeutic approach because of their unique mechanism of action in combating therapy-resistant cancer cells. Notably, metamizole has been shown to display inhibitory effects on GPX4 in vitro [33]. Taken together, the previously mentioned cell line studies and the findings from our study suggest that, in addition to non-apoptotic pathways such as ferroptosis, metamizole may also activate classical apoptotic mechanisms.

Metamizole is pre-systemically converted to 4-MAA in the intestine and/or liver. 4-MAA has a high oral bioavailability of greater than 80% and is the major metabolite in plasma [34]. In a study conducted to investigate the pharmacokinetics of metamizole, the drug was administered orally to healthy volunteers in single doses of 750, 1500, and 3000 mg. Following administration, 4-MAA Cmax levels were determined as 10.6, 20.5, and 41.4 µg/mL (approximately 48.33, 94.35, 190.5 µM—Molecular weight of 4-MAA: 217.27 g/mol), respectively [35]. Although metabolite concentrations were not measured in our study, the concentrations used were within a range that could encompass these plasma levels. Unlike in vivo studies, in cell culture, the effect remained constant at these concentrations, as pharmacokinetic influences are minimal in cell culture models. However, it should not be forgotten that the drug is used in repeated doses for treatment of chronic diseases.

**There are several limitations to this study.** In the study, ELISA was performed only for caspase-3, which is involved in apoptotic pathways. In addition, in order to show apoptotic effects, only fold changes in Bax and Bcl-2 mRNA expression were analyzed using RT-qPCR. We addressed these limitations by assessing apoptotic cell percentages via flow cytometry, as well as proliferation and mitotic indices. Normal human cell lines were not used to evaluate the safety profile of metamizole, but in our previous studies, we obtained results about its effects on hematopoietic progenitor cells obtained from healthy bone marrow donors. We selected K562 cells based on our prior findings indicating metamizole’s effects on myeloid-derived cells, and its association with agranulocytosis as a known adverse effect. We designed and completed our study in this direction within the constraints of available resources.

## Conclusion

Our results were obtained through complementary analytical methods and it was determined for the first time that metamizole can induce apoptosis in K562 cells. It also showed that mitotic cell ratio analysis, which is generally used in different cellular-based studies, may also be useful tool in drug effect studies and contribute to the interpretation of the results. In our experiments, the number of cells in the mitosis stage decreased toward higher concentrations but remained detectable. In addition, the necrotic cell ratio remained largely unchanged, except at 100 µM concentration. Bax, Bcl-2 mRNA expressions, and caspase-3 concentrations did not differ significantly at either 24 or 48 h across increasing concentrations. These results indicate that metamizole reduces proliferation via apoptotic mechanisms rather than through severe cytotoxicity. The irreversible transformation of early apoptotic cells at 24 h of exposure to apoptotic cells at 48 h of exposure indicates that metamizole probably affects at a certain proportion of cells at the first contact. Further in vivo studies with repeated dosing are required for more definitive conclusions. NSAIDs have been frequently analyzed for different indications in recent years. Cancer treatment is often complex and costly. Identifying the potential antitumor effects of metamizole and other NSAIDs may support their integration into combination therapies with conventional antineoplastics. Especially if its hematopoietic side effects are better understood through mechanistic studies, it may be an alternative in the treatment of myeloid cell-based malignancies.

## Supplementary Information

Below is the link to the electronic supplementary material.

Supplementary file1 (PDF 412 KB)

## Data Availability

No datasets were generated or analyzed during the current study.
