# Supplementary material for: Metamizole limits proliferation in chronic myeloid leukemia cells and triggers apoptosis via the bax/bcl-2/caspase-3 cascade
Source: Med Oncol. 2025 Jun 27;42(8):288. doi: 10.1007/s12032-025-02842-x (PMC12204905; doi:10.1007/s12032-025-02842-x)
Supplement: Supplementary file 1 — Supplementary file1 (PDF 412 KB) [file 12032_2025_2842_MOESM1_ESM.pdf]

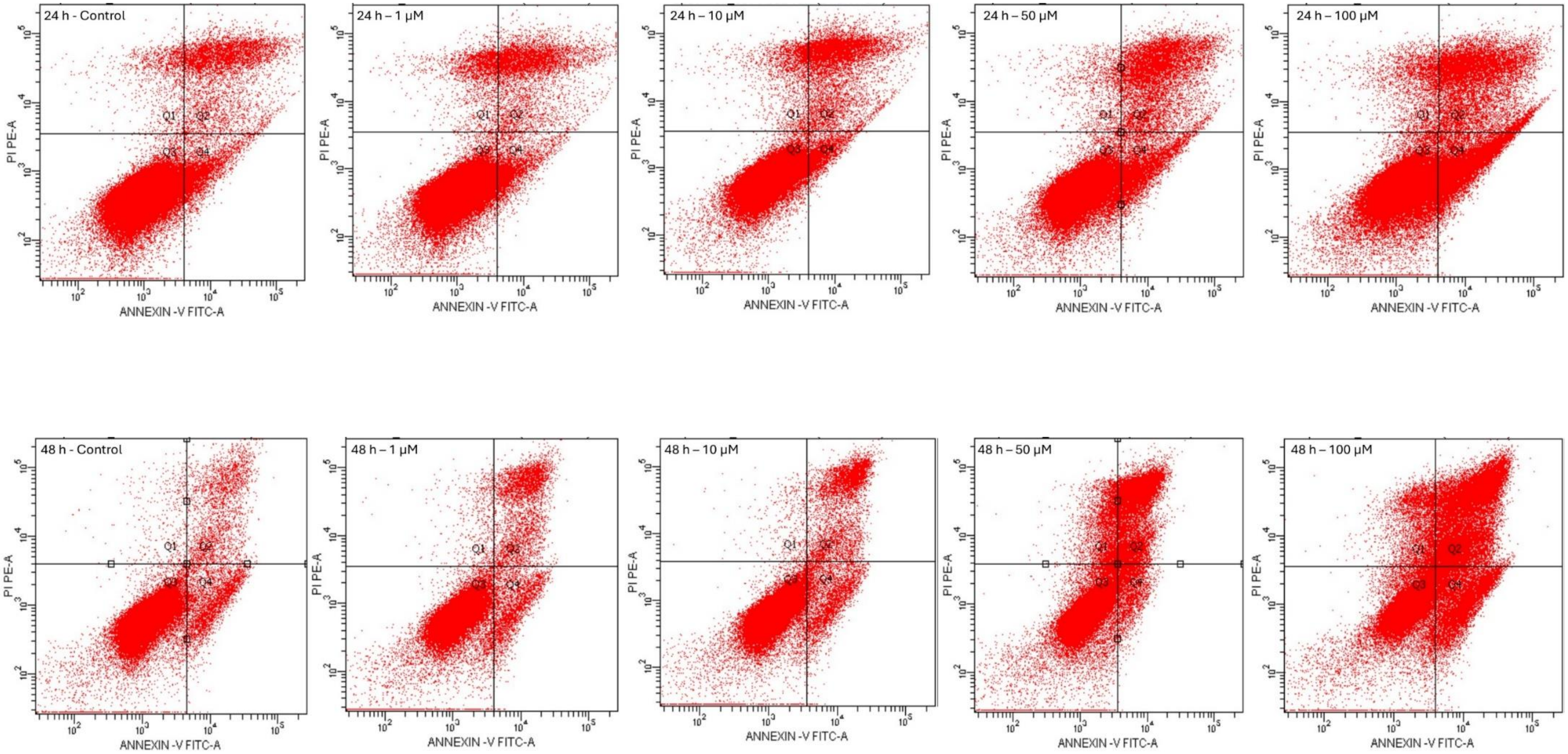

Figure Suppl: Healthy (Q3), early apoptotic (Q4), apoptotic (Q2) and necrotic (Q1) cells in flow cytometry images according to metamizole concentrations and time.
